# Effects of radial versus femoral artery access in patients with acute myocardial infarction: A large centre prospective registry

**DOI:** 10.1007/s12471-016-0887-6

**Published:** 2016-08-25

**Authors:** S. Kilic, R. S. Hermanides, J. P. Ottervanger, E. Kolkman, J. H. E. Dambrink, V. Roolvink, A. T. M. Gosselink, E. Kedhi, A. W. J. van ’t Hof, Zwolle Myocardial Infarction Study Group

**Affiliations:** Department of Cardiology, Isala Klinieken Zwolle, Zwolle, The Netherlands

**Keywords:** Radial artery access, NSTEMI, STEMI, Primary percutaneous coronary intervention

## Abstract

**Aim:**

This study sought to assess whether radial artery access improves clinical outcomes in patients presenting with acute myocardial infarction compared with femoral artery access.

**Methods:**

This is a single-centre, prospective observational registry of all STEMI and NSTEMI patients who underwent coronary angiography and/or primary PCI in the period January 2010 to December 2013. Primary endpoint was 30-day all-cause mortality. Choice of access was left to the discretion of the cardiologist. Differences in the risk of death at 30 days between patients undergoing transradial intervention versus transfemoral intervention was assessed on an intention-to-treat comparison.

**Results:**

Retrospective analysis of prospectively collected data was performed in 3580 patients with an acute coronary syndrome who underwent coronary angiography, of which 1310 had radial artery access. PCI was performed in 77 % of the patients. Before propensity score matching, patients who underwent transradial intervention and those intended to undergo transfemoral approach differed significantly in intra-aortic balloon pump use (1.7 % vs. 6.7 %, *p* < 0.001), and Killip class (Killip 1: 10.8 % vs. 17.3 %, *p* < 0.001). 30-day mortality rates were 1.7 % in the transradial group and 4.6 % in the transfemoral group (*p* < 0.001). After matching on the propensity score, the hazard ratio for 30-day mortality in the transradial group was 0.56 (95 % CI: 0.29–1.07, *p* = 0.08).

**Conclusion:**

This registry-based study showed that radial access is associated with improved outcome in patients with an acute coronary syndrome. However, this difference was no longer significant after multivariate and propensity score adjustment for differences in baseline characteristics.

## Introduction

In patients with acute coronary syndrome (ACS), early and complete restoration of blood flow has been shown to improve long-term outcomes [1, 2]. For both diagnostic coronary angiography and percutaneous coronary intervention (PCI) a transradial approach to vascular access (transradial intervention) is rapidly becoming preferable to traditional transfemoral intervention [3, 4]. Myocardial infarction (MI) and PCI-related bleeding have been strongly associated with early and late mortality [5–9]. The use of radial access has been demonstrated to be feasible in the ACS setting and, compared with femoral access, a reduction in vascular complications and bleeding has been suggested [10, 11]. Whether this evident reduction in access-site bleeding may also have a positive impact on prevention of further cardiovascular events remains to be defined. The available clinical evidence summarised in a recent meta-analysis seems to suggest that the radial approach could also be associated with improved outcome [12]. It is possible that mortality and ischaemic events may also be reduced by this technique. The primary aim of this observational study was to evaluate the effect of radial artery access on 30-day all-cause mortality in an unselected all-comer ST-segment elevation myocardial infarction (STEMI) and non-ST-segment elevation myocardial infarction (NSTEMI) population who were undergoing coronary angiography in a high-volume cardiothoracic centre.

## Methods

All consecutive STEMI and NSTEMI patients undergoing coronary angiography at Isala, Zwolle in the Netherlands between January 2010 and December 2013 were included. STEMI patients were defined as those presenting with ischaemic symptoms >30 min with ST-segment elevation of >2 mm in two contiguous precordial leads or >1 mm in two contiguous limb leads or new left bundle branch block. NSTEMI was defined by the presence of ischaemic chest pain (or another complaint suggestive of ischaemia, such as shortness of breath of collapse), the notable absence of ST-segment elevation on electrocardiography, and the presence of either ST-segment depression or T‑wave inversion on electrocardiography and/or elevated cardiac biomarkers. All STEMI patients were directly transported to the catheterisation laboratory on arrival, and acute coronary angiography was performed with subsequent primary PCI when indicated. All NSTEMI patients were treated according to the current NSTEMI-ACS guidelines [13]. The decision to use radial or femoral access was at the discretion of the treating cardiologist. Patients who had a crossover of access were excluded from analysis. All patients were pre-treated with aspirin, heparin, and clopidogrel (600 mg loading dose), or ticagrelor (180 mg loading dose) during transportation to the hospital, or these drugs were administered in the emergency room. The use of glycoprotein (GP) IIb/IIIa inhibitors or bivalirudin was left to the operator’s discretion. There were no exclusion criteria with regard to age, sex, ischaemic time, cardiac history, or renal failure.

### Study design

This was a prospective observational cohort study. Baseline demographics, clinical presentation, procedure details and procedural complications were collected by the performing physician at the end of each procedure. Post-procedural complications, clinical data and discharge medications were updated on discharge. Follow-up data of mortality during index hospital admission (in-hospital) and 30-day mortality were collected at the routine outpatient visit or by telephone interview of the patient by research personnel. The primary outcome measure was 30-day all-cause mortality.

Major bleeding was defined as either intracranial bleeding or overt bleeding with a decrease in haemoglobin ≥5 g/dl (≥3.1 mmol/l) or a decrease in haematocrit ≥15 % within 30 days after admission. Minor bleeding was defined as identified bleeding with a decrease in haemoglobin ≥3 g/dl (≥1.9 mmol/l), or >10 % decrease in haematocrit [14].

### Statistics

Because the patients were not randomly assigned to undergo transradial intervention, a propensity score analysis was performed by using a logistic regression model for transradial versus transfemoral intervention to adjust for differences in baseline characteristics [15]. This analysis included a number of clinical, angiographic, and procedural variables: age, sex, hypertension, STEMI, out-of-hospital cardiac arrest (OHCA), ischaemic time (onset of symptoms to angiogram), year of procedure, previous coronary artery bypass grafting (CABG), PCI performed, P2Y12 inhibitors during acute phase, heparin during acute phase, GP IIb/IIIa inhibitors during acute phase, intra-aortic balloon pump (IABP), Killip >1, previous PCI, family history, previous MI, previous cerebrovascular accident. Patients in the transradial group were matched to patients in the transfemoral group with the closest propensity score. Only pairs of patients in which the difference between propensity scores was <0.2 were selected. After all the propensity score matching was performed, we compared the baseline covariates between the two intervention groups. Continuous variables were compared using the paired T‑test or the Wilcoxon signed-rank test, as appropriate, and categorical variables were compared using McNemar’s test. The statistical significance and the effect of treatment on outcomes were estimated using appropriate statistical methods for matched data. In the propensity score-matched cohort, the risks of each outcome were compared using Cox regression models which accounted for the clustering of matched pairs. Survival curves were also constructed with Kaplan-Meier estimates and compared by the Klein-Moeschberger test. Also the propensity score that was generated in the whole patient population was incorporated into subsequent proportional hazards models as a covariate. To avoid over-adjustment, the multivariable Cox regression analysis was performed using only the two variables ‘propensity score’ and ‘treatment.’ All reported *p* values are two-sided, and *p* values of <0.05 were considered to indicate statistical significance. IBM SPSS software (version 20, SPSS Inc, Chicago, USA) was used for statistical analyses.

## Results

A total of 3580 STEMI and NSTEMI patients were studied. The percentage of patients undergoing transradial intervention increased from 19 % in 2010 to 74.5 % in 2013. The rate of crossover from primarily intended transradial to transfemoral intervention was 4 % in 2010, to 3 % in 2011, 2.2 % in 2012 and 4.6 % in 2013. Baseline, clinical and angiographic characteristics are shown in Table 1 and 2.

**Table 1:** Demographics and clinical characteristics

	**Radial** **(** ***n*** **= 1310)**	**Femoral** **(** ***n*** **= 2270)**	***p*** ** value**
Age (years)	65.41 ± 12.74	64.75 ± 12.62	0.172
Male gender	906/1310 (69.2)	1613/2270 (71.1)	0.231
Hypertension	620/1294 (47.9)	1006/2264 (44.4)	0.045
Diabetes mellitus	204/1296 (15.7)	318/2266 (14.0)	0.166
Smoke	457/1290 (35.4)	817/2257 (36.2)	0.645
Hypercholesterolaemia	327/1251 (26.1)	579/2155 (26.9)	0.643
Family history	448/1281 (35.0)	827/2261 (36.6)	0.339
Previous MI	179/1297 (13.8)	293/2265 (12.9)	0.464
Previous PCI	188/1298 (14.5)	311/2265 (13.7)	0.533
Previous CABG	68/1298 (5.2)	171/2265 (7.5)	0.008
Previous CVA	39/1298 (3.0)	69/2266 (3.0)	0.946
STEMI	796/1310 (60.8)	1390/2270 (61.2)	0.781
PCI performed	1033/1308 (79.0)	1701/2267 (75.0)	0.007
GPI in acute phase	376/1299 (28.9)	752/2254 (33.4)	0.006
IABP	21/1262 (1.7)	148/2240 (6.6)	<0.001
Killip class >1	138/1274 (10.8)	391/2263 (17.3)	<0.001
OHCA	35/781 (4.5)	30/781 (3.8)	0.17

Values are number of cases (%) or mean ± standard deviation

*MI* myocardial infarction, *PCI* percutaneous coronary intervention, *CABG* coronary artery bypass grafting, *CVA* cerebrovascular accident, *STEMI* ST-segment elevation myocardial infarction, *IABP* intra-aortic balloon pump, *GPI* glycoprotein IIb/IIIa inhibitor, *OHCA* out-of-hospital cardiac arrest

**Table 2:** Angiographic characteristics

	**Radial** **(** ***n*** **= 1310)**	**Femoral** **(** ***n*** **= 2270)**	***p*** ** value**
*Initial TIMI flow*	*–*	*–*	*0.063*
0	417/1077 (38.7)	790/1867 (42.3)	–
1	72/1077 (6.7)	141/1867 (7.6)	–
2	121/1077 (11.2)	220/1867 (11.8)	–
3	467/1077 (43.4)	716/1867 (38.4)	–
*TIMI post PCI*	*–*	*–*	*0.753*
0	12/991 (1.2)	23/1683 (1.4)	–
1	7/991 (0.7)	12/1683 (0.7)	–
2	36/991 (3.6)	75/1683 (4.5)	–
3	936/991 (94.5)	1573/1683 (93.5)	–
PCI	1033/1308 (79.0)	1701/2267 (75.0)	0.007
CABG	86/1308 (6.6)	192/2270 (8.5)	0.444
No revascularisation	191/1310 (14.6)	377/2270 (16.6)	0.110
IRA	–	–	0.035
LAD	442/1084 (40.8)	781/1995 (39.1)	–
CX	23/1084 (21.3)	401/1995 (20.1)	–
RCA	367/1084 (33.9)	679/1995 (34)	–
Graft	29/1084 (2.7)	77/1995 (3.9)	–
LM	15/1084 (1.4)	57/1995 (2.9)	–

Values are number of cases (%)

*TIMI* thrombolysis in myocardial infarction, *PCI* percutaneous coronary intervention, *CABG* coronary artery bypass grafting, *IRA* infarct-related artery, *LAD* left anterior descending artery, *CX* circumflex artery, *RCA* right coronary artery, *LM* left main artery

### Unadjusted outcomes

Before propensity score matching, patients who underwent transfemoral intervention and those intended to undergo the transradial approach differed significantly in clinical and procedural characteristics (Table 1). Patients with previous CABG (5.2 % in the transradial vs. 7.5 % in the transfemoral group; *p* = 0.008), GP IIb/IIIa inhibitors in the pre-hospital phase (28.9 % in the transradial vs. 33.4 % in the transfemoral group; *p* = 0.006), patients with IABP (1.7 % in the transradial vs. 6.6 % in the transfemoral group; *p* < 0.001) and patients with Killip class >1 (10.8 % in the transradial vs. 17.3 % in the transfemoral group; *p* < 0.001) were more likely to be treated by transfemoral intervention. The unadjusted rate of death in the first 30 days after treatment was lower among patients undergoing transfemoral intervention (1.7 % in the transradial vs. 4.6 % in the transfemoral group; *p* < 0.001). There was no significant difference in MI (0.6 % in the transradial vs. 0.9 % in the transfemoral group; *p* = 0.216) and stroke (0 % in the transradial vs. 0.3 % in the transfemoral group; *p* = 0.093). The rate of overall bleeding was significantly reduced in the transradial group at 30 days (10.9 % vs. 13.6 % in the transfemoral group; *p* = 0.02). The rate of major bleeding was also significantly reduced in the transradial group at 30 days (4.7 % vs. 7.8 % in the transfemoral group; *p* = 0.001). There was no significant difference in minor bleeding between the transradial and the transfemoral group (6.2 % in the transradial vs. 5.8 % in the transfemoral group; *p* = 0.643).

### Propensity score-adjusted outcomes

**Fig. 1:**
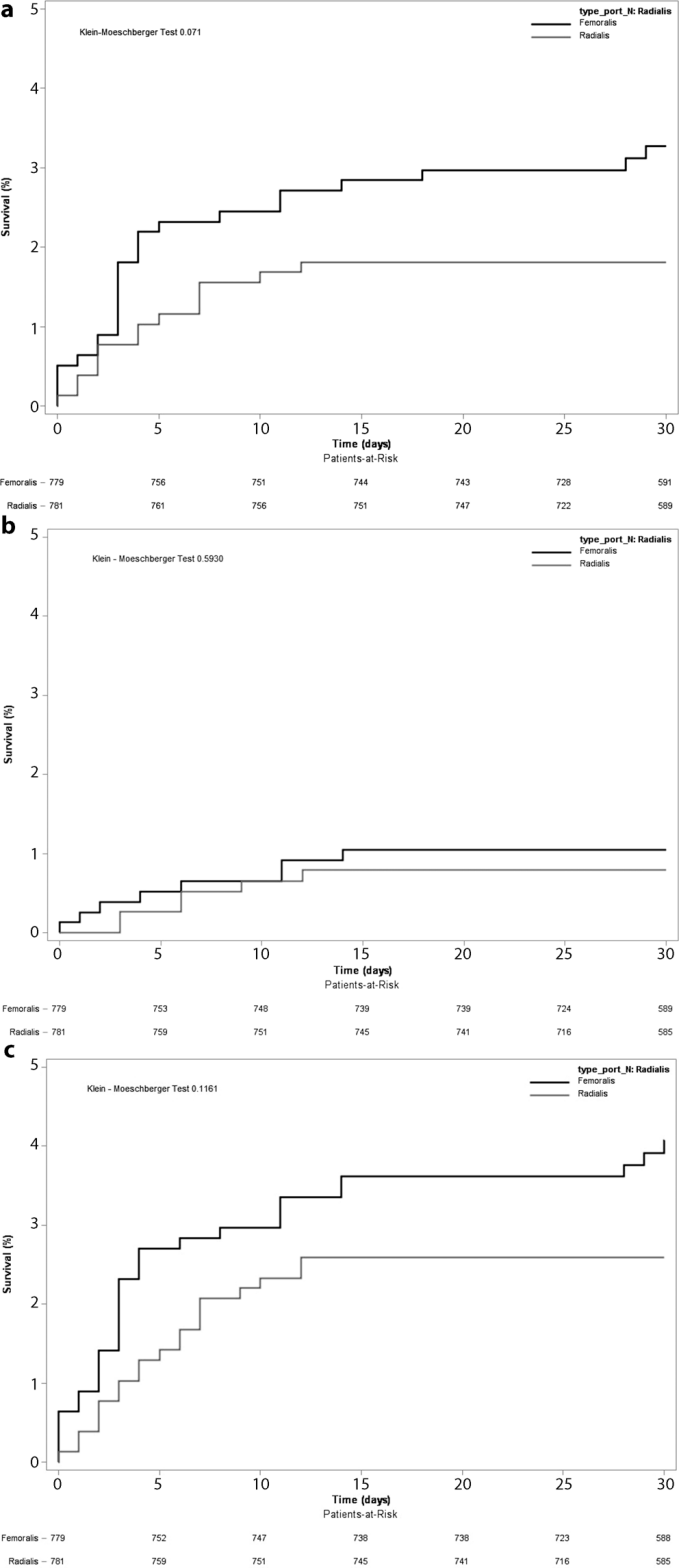
Kaplan-Meier curves for outcomes in the propensity score-matched cohort of patients. Patients who underwent transradial or transfemoral intervention were matched for propensity scores. Propensity matching for the entire cohort created 781 matched pairs of patients. (**a**) Outcomes for overall 30-day mortality; (**b**) outcomes for myocardial infarction; (**c**) outcomes for 30-day mortality, myocardial infarction, or stroke

After propensity score matching was performed, there were 781 matched pairs of patients (Table 3). The adjusted outcomes based on propensity score analysis showed no significant difference for mortality (HR 0.56 95 % CI [0.29–1.07], *p* = 0.08) at 30 days in both groups (Fig. 1a). There was also not a significant difference for MI (HR 0.75 95 % CI [0.26–2.15], *p* = 0.59) (Fig. 1b) and for composite death, MI, and stroke (HR 0.64 95 % CI [0.37–1.13], *p* = 0.12) (Fig. 1c). Major bleeding (HR 0.61 [0.25–1.47], *p* = 0.27) and minor bleeding (HR 0.75 [0.32–1.77], *p* = 0.51) showed no significant differences. Although not statistically significant, there was a trend towards a favourable outcome for transradial intervention for all endpoints.

**Table 3:** Characteristics of the propensity score-matched patients

	**Femoral** **(** ***n*** **= 781)**	**Radial** **(** ***n*** **= 781)**	***p*** ** value**
STEMI (%)	454/781 (58.1)	461/781 (59.0)	0.69
Male gender (%)	548/781 (70.2)	550/781 (70.3)	0.91
Age (years)	65.38 ± 12.77	65.14 ± 12.30	0.66
Hypertension (%)	364/781 (46.6)	363/781 (46.5)	0.96
Diabetes mellitus (%)	100/780 (12.8)	116/780 (7.4)	0.24
Smoking (%)	277/780 (35.5)	274/779 (35.2)	0.87
Hypercholesterolaemia (%)	200/747 (26.8)	195/748 (26.1)	0.76
Family history (%)	288/781 (36.9)	277/781 (35.5)	0.56
Previous MI (%)	108/781 (13.8)	110/781 (14.1)	0.88
Previous PCI (%)	114/781 (14.6)	111/781 (14.2)	0.83
Previous CABG (%)	54/781 (3.5)	51/781 (3.3)	0.72
Previous CVA (%)	22/781 (2.8)	21/781 (2.7)	0.88
PCI performed (%)	617/781 (79)	618/781 (79)	0.95
IABP (%)	19/781 (2.4)	17/781 (2.2)	0.71
Killip >1 (%)	88/781 (11.3)	94/781 (12.0)	0.60
OHCA (%)	29/781 (3.7 %)	35/781 (4.5)	0.37
P2Y12 in acute phase (%)	693/781 (88.7)	687/781 (88.0)	0.62
Heparin in acute phase (%)	579/781 (74.1)	587/781 (75.2)	0.64
GPI in acute phase (%)	240/781 (30.7)	246/781 (31.5)	0.73

Values are number of cases (%) or mean ± standard deviation

*MI* myocardial infarction, *PCI* percutaneous coronary intervention, *CABG* coronary artery bypass grafting, *CVA* cerebrovascular accident, *IABP* intra-aortic balloon pump, *OHCA* out-of-hospital cardiac arrest, *GPI* glycoprotein IIb/IIIa inhibitor

**Fig. 2:**
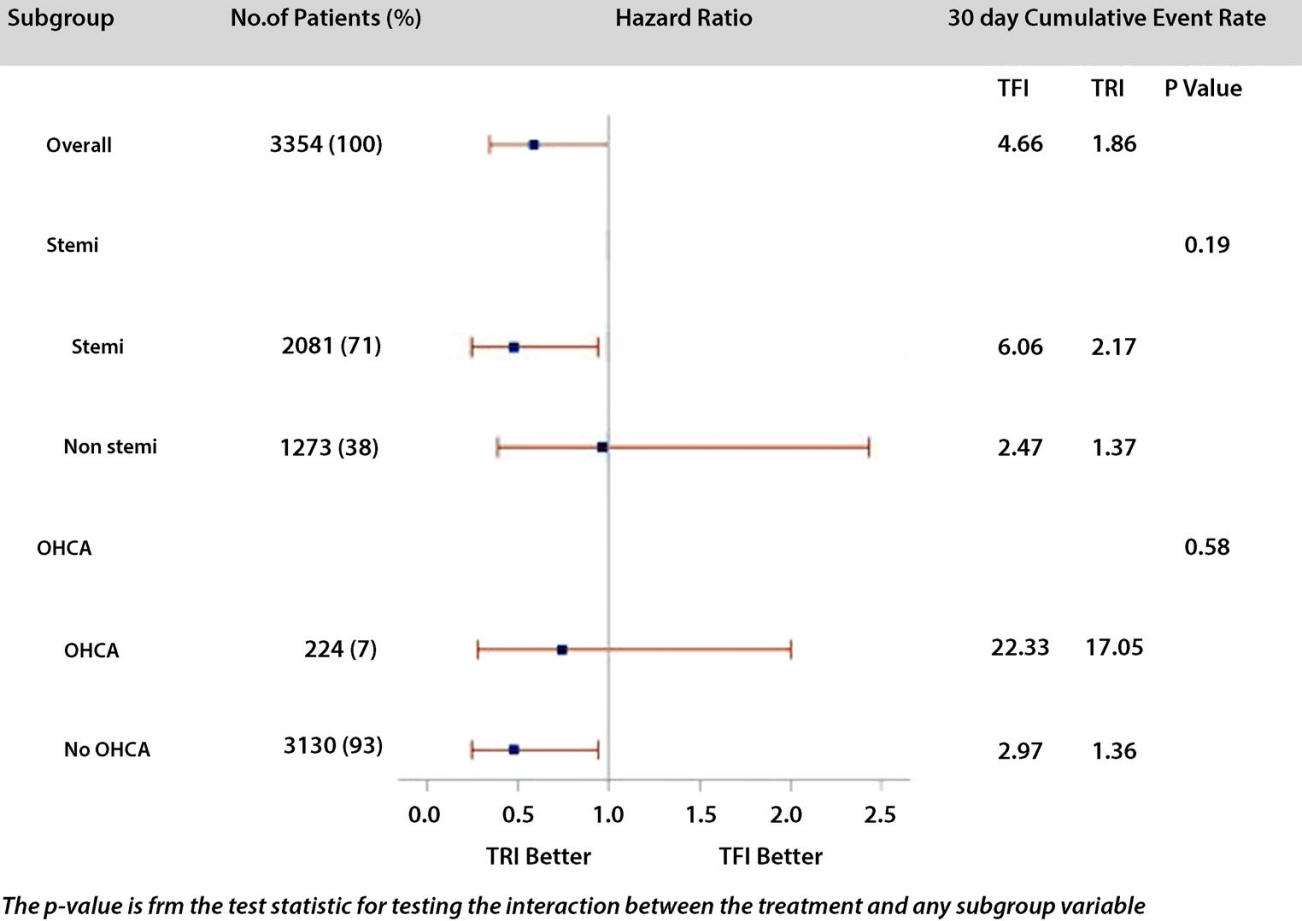
Subgroup analysis for 30-day mortality showed for transfemoral vs. transradial intervention in the STEMI vs. NSTEMI and OHCA vs. no-OHCA

A subgroup analysis comparing STEMI vs. NSTEMI patients showed that the transradial approach was associated with significantly lower mortality at 30 days in STEMI patients (1.7 % in the transradial vs. 5.6 % in the transfemoral group; *p* < 0.001) and a non-significant difference in NSTEMI patients (1.6 % in the transradial vs. 2.2 % in the transfemoral group; *p* = 0.47). A subgroup analysis comparing OHCA vs. no-OHCA patients showed that that transradial intervention was associated with significantly lower mortality at 30 days in no-OHCA patients (transradial: 1.3 % vs. transfemoral: 2.7 %, *p* = 0.009), and a non-significant difference in OHCA patients (transradial: 14.3 %, transfemoral: 21.3 %, *p* = 0.34). There was no significant interaction between the type of MI, OHCA and vascular access site, (Fig. 2).

## Discussion

This prospective observational cohort study, based on a large and unselected cohort of patients, showed that transradial intervention was associated with reduced mortality at 30 days; however, this difference was no longer significant after multivariate and propensity score adjustment. There was a trend towards a favourable outcome for the transradial approach for all endpoints. Transfemoral intervention is currently considered the gold standard access site worldwide [3]; however, access-site complications remain frequent in clinical practice. Theoretically, transradial intervention may improve the survival rate by minimising access site-related complications and their negative prognostic consequences [16]. It would be difficult to reduce bleeding complications in a setting of patients with high thrombotic risk where aggressive antithrombotic and antiplatelet therapy drives gastrointestinal, intracranial and other non-access-site bleeding [17]. Transradial intervention may be underutilised in high-risk ACS patients who may derive greater benefit from this approach. The RIVAL (Radial Versus Femoral Access for Coronary Intervention) study was a large randomised trial of radial and femoral artery access in patients with ACS. It failed to show a significant reduction in death, MI, or bleeding despite significant reductions in vascular access complications. In the subgroup of STEMI patients, however, transradial intervention significantly reduced the primary outcome of death, MI, or stroke and secondary outcomes mainly by a reduction in mortality with a directionally consistent reduction in MI [10, 18]. The RIFLE-STEACS was a large randomised clinical trial comparing the radial and femoral approaches performed in patients with STEMI only and showed that radial access was associated with significantly lower morbidity and cardiac mortality [19]. Subgroup analysis in our study showed no significant interaction in clinical outcome between STEMI vs. NSTEMI for patients treated with transradial vs. transfemoral intervention. The recently published MATRIX trial (Minimising Adverse Haemorrhagic Events by Transradial Access Site and Systemic Implementation of Angiox) randomised 8404 patients undergoing angioplasty. This large trial included STEMI and NSTEMI patients. After 30 days, the use of radial access for coronary angiography followed by percutaneous coronary intervention, if indicated, significantly reduced the rate of net adverse clinical events, defined as the composite of major adverse cardiovascular events or major bleeding [20]. Systematic reviews have repeatedly demonstrated that transradial intervention in unselected populations for coronary angiography resulted in an absolute reduction in bleeding complications, leading to reduced ischaemic endpoints and death [21]. Especially in high-risk populations, such as patients with extreme obesity, reductions resulted in an absolute reduction in vascular complications [22, 23]. Selecting a transradial approach for coronary angiography or PCI may be an important intervention for reducing procedural morbidity in patients considered at high risk for access site complications. The radial access route is still not widely utilised despite current evidence. The radial approach is a specific skill and involves a learning curve [24, 25]. There are a few known complications, such as upper extremity dysfunction after transradial intervention, radial artery spasm, intimae damage and occlusion, or vascular anomalies with consequent failure to reach the ascending aorta. These are obstacles that can be overcome with appropriate training [11, 26, 27].

### Limitations

Several limitations of the present analysis should be considered. First, this is a single-centre, non-randomised, prospective observational study and therefore it was not possible to account for all confounding influences. We used propensity score matching to make the patient groups comparable according to the measured confounders, and we successfully eliminated the observed differences. However, we cannot fully exclude the possibility of confounding by baseline factors that we did not study. Second, we also could not control for differences in the skill of the operators. It is conceivable that a radial approach may be preferentially selected by more skilled angiographers.

## Conclusion

In this single-centre registry, the use of radial access for all consecutive STEMI and NSTEMI patients undergoing coronary angiography and PCI when appropriate showed that radial access was associated with improved outcome. However, this difference was no longer significant after multivariate and propensity score adjustment for differences in baseline characteristics.
